# Case of Gonorrhœa, Produced by Swallowing Gonorrhœal Discharge

**Published:** 1833-09

**Authors:** 


### ORIGINAL PAPERS.

### GONORRHŒA.

### *Case of Gonorrhœa, produced by swallowing Gonorrhœal Discharge.* By Dr. Tazentre.

[We will not take upon ourselves to determine the degree of credit which our readers may give to the inferences drawn from this extraordinary, and to us quite novel, case. The fidelity of the relator we can have no right to question; but we confess we are inclined to suspect that some deception was used, or that some more ordinary cause than that assigned produced the gonorrhœal symptoms in the female upon whom the disgusting *ruse* was practised. Upon these points, however, we leave our readers to form their own opinion. The following is a correct account of Dr. Tazentre's curious detail, from the *Archives Génèrales,* Juin 1833.]

In the inaugural dissertation which he defended at the Faculty of Medicine at Paris, Dr. Tazentre laid down the following proposition: "Gonorrhœal matter taken into the stomach, and the *dose!* repeated for several days, may produce syphilitic inflammation of the urethra." This opinion was founded upon the following very remarkable fact, which we are assured is faithfully and exactly given.

M. N., fifty-five years of age, an old sailor of licentious habits, was married to a young woman of twenty. The matrimonial tie did not, however, confine him from drunkenness and debauchery. About three years after his marriage he suspected that his wife played him false, but he had no proof that his suspicions were well founded. Having contracted a very severe gonorrhœa from one of his mistresses, he was anxious to cohabit with his wife, with whom he had had no connexion for two or three months, in order that he might communicate the disease to her, and then accuse her of having infected him. By this stratagem he hoped to extract from her a confession of her frailty. But she had observed that he looked unwell, and appeared to be suffering for many days, and she consequently refused the marital privilege. Baffled in this attempt, he had recourse to another expedient: he gave her secretly, mixed in milk or orgeat, a quantity of the gonorrhœal discharge, which he collected in a glass. He played this disgusting trick for eight or ten days, when one morning his helpmate unexpectedly entered the room, and detected him in the act of mixing something white with the milk that she had left upon the table for her breakfast. She accused him of doing something wrong, and declared she would have the milk analysed by a chemist. Alarmed at her threat, he confessed his perfidy. The woman herself was very naturally apprehensive that injurious effects might arise from the sort of poison she had taken, and she communicated the whole story to her parents.

Dr. Tazentre was acquainted with the family, and he was consulted upon the occasion. As yet Madame N. complained of no pain in the genital organs, nor was any discharge or inflammation detected, upon an examination made by Dr. T. himself. As he could not anticipate what phenomena might result from this new mode of infection, he merely prescribed a light and temperate diet, and occasional warm baths. Four days afterwards the patient again applied to him, complaining of pains in the vagina. He again examined her, and found the parts tumid and painful; and around the urethra there was considerable inflammation. On the following days the pain, particularly in making water, became much more severe, and the discharge more abundant. She had, in fact, all the symptoms of virulent gonorrhœa. A small reddish-looking streak extended from the clitoris to the groin, the glands of which were painful.

As the nature of the case was no longer doubtful, an appropriate treatment was adopted, and in about a week mercury was given internally, as Dr. T. was convinced of the existence of constitutional disease. Under the influence of this treatment Madame N. soon perfectly recovered.

Having been entrusted with the secret of the family, Dr. Tazentre's assistance was asked for in the husband's case. He also recovered from his gonorrhœa in about three weeks. Dr. T. obtained from him a particular account of the whole circumstances of the above related facts. He confessed every thing without reserve, and said that he had been anxious to communicate gonorrhœa to his wife, without exciting her suspicions, in order to force her to a disclosure of her infidelity. He added, that he was sure of infecting her by the means he adopted; for that formerly, wishing to revenge himself upon some person against whom he had a grudge, he had given him syphilis complicated with gonorrhœa, by administering, in the manner above mentioned, gonorrhœal discharge; and that it was in the colonies, where such tricks he declared were occasionally resorted to, that he had learned this mode of poisoning.

Dr. T. remarks, that this curious and unique fact, if confirmed by others of a similar nature, would be important in medical jurisprudence. It proves, in his opinion, the specific properties of gonorrhœal matter; and completely overthrows the doctrine which teaches us to regard gonorrhœa as a local inflammatory disease, which is always to be cured by a local antiphlogistic treatment. If the gonorrhœal matter is but the product of simple inflammation of the mucous membrane lining the urethra, how could it produce the disease without being placed in immediate contact with the part? If it were simply irritating, and not possessing any specific property, how could it traverse the digestive organs without injury to them, and localize itself and exert its action upon the urethra alone? The above case shews that there is a very different but unknown kind of affinity between the discharge and the genital organs, which must be regarded as specific.

Practitioners whose theory is opposed to this view may object to the facts which have been stated. It may be said that Madame N. might have contracted gonorrhœa from the person with whom her husband suspected her of intriguing, or that she merely laboured under profuse leucorrhœa, which Dr. T. mistook for gonorrhœa. He did not overlook these objections in collecting information upon the case, and he was therefore doubly cautious in his inquiries. The account he gives he received from the parties themselves, and it cannot be supposed that they exaggerated the facts for the purpose of rendering themselves more culpable. The man with whom Madame N. had connexion (for the suspicions of her husband were well founded,) was not affected with gonorrhœa. Dr. T. had undeniable proof of this fact: from him, therefore, her disease could not have originated. It was equally impossible for her to be deceived as to the nature of the disease. The severe pain she suffered in making water; the seat of the inflammation; the abundant discharge, thick and whitish at first, and subsequently of a greenish colour; the obstinacy of the inflammation; the red cord running from the clitoris to the groin, the glands of which became painful, form a series of symptoms that could not be mistaken for leucorrhœa; to which, besides, Madame N. had never been subject. Lastly, Dr. T. was persuaded that secondary symptoms would have occurred if he had not adopted the proper and precautionary practice.

An interesting note is appended to the original paper, in which Dr. T. anticipates some objections that may be made to this case. He presumes that the absorbing power of the mucous surfaces will not be denied; but it may be replied, that, in the above case, absorption must have taken place in the stomach, and that experiments upon morbid poisons appear to prove that their action is neutralised by the action of the stomach. Proofs are yet wanting, however, to establish this position. Fontana made many experiments upon the poison of certain animals, particularly upon that of the viper: he has shewn that the venom of this reptile, taken into the stomach, produces no sensible effects, and that, when applied to the eye, it merely caused a slight degree of redness. He had even the courage to place it upon his own and his servant's tongue; and the only sensation experienced was a sweetish taste, and a slight feeling of constriction. Such experiments, frequently repeated, led to the inference that, provided the mucous surface of the mouth and tongue was not abraded, there was no absorption of the poison. These are conclusive experiments as far as regards the poison of the viper; but they go no further. Dr. T. thinks we should commit a serious mistake if we, from these facts, formed similar conclusions respecting all other poisons. If Fontana had experimented upon the serpent, whose venom is more deleterious, he would probably have obtained poisonous effects; for M. Rousseau experimented some years ago upon the rattlesnake, and he found that its poison was absorbed by mucous surfaces, and that animals died into whose eyes it had been inserted. It is to be regretted that M. Rousseau did not introduce the poison into the stomach of an animal; for probably, if he had done so, its destructive power would not have been neutralised by the gastric action. The term virus, created by pathologists to express a particular germ, producing in the system to which it is applied special and constant symptoms, and always the same for each virus, is nothing more than an abstraction which includes the complex ideas of very different products. It has tended to group under one name morbid stimuli, the essence of which is unknown, and whose mode of action is very different. Each of them has peculiar properties, and produces peculiar effects, and therefore no general conclusion can be derived from experiments formed with any one singly. They must be studied and experimented upon separately.

